# Transcriptomic and Metabolomic Profiling Reveals the Variations in Carbohydrate Metabolism between Two Blueberry Cultivars

**DOI:** 10.3390/ijms25010293

**Published:** 2023-12-25

**Authors:** Haiyan Yang, Zhiwen Wei, Yaqiong Wu, Chunhong Zhang, Lianfei Lyu, Wenlong Wu, Weilin Li

**Affiliations:** 1 Jiangsu Key Laboratory for the Research and Utilization of Plant Resources, Institute of Botany, Jiangsu Province and Chinese Academy of Sciences (Nanjing Botanical Garden Mem. Sun Yat-Sen), Nanjing 210014, China; haiyanyang@cnbg.net (H.Y.); ya_qiong@126.com (Y.W.); chzhang@cnbg.net (C.Z.); njbglq@163.com (L.L.); 2 Co-Innovation Center for Sustainable Forestry in Southern China, College of Forestry, Nanjing Forestry University, Nanjing 210037, China; wzw0709wzw@163.com

**Keywords:** blueberry, fruit quality, sugar accumulation, starch and sucrose metabolism, glycolysis/gluconeogenesis, pentose phosphate pathway

## Abstract

Blueberry is a high-quality fruit tree with significant nutritional and economic value, but the intricate mechanism of sugar accumulation in its fruit remains unclear. In this study, the ripe fruits of blueberry cultivars ‘Anna’ and ‘Misty’ were utilized as experimental materials, and physiological and multi-omics methodologies were applied to analyze the regulatory mechanisms of the difference in sugar content between them. The results demonstrated that the ‘Anna’ fruit was smaller and had less hardness than the ‘Misty’ fruit, as well as higher sugar content, antioxidant capability, and lower active substance content. A total of 7067 differentially expressed genes (DEGs) (3674 up-regulated and 3393 down-regulated) and 140 differentially abundant metabolites (DAMs) (82 up-regulated and 58 down-regulated) were identified between the fruits of the two cultivars. According to KEGG analysis, DEGs were primarily abundant in phenylpropanoid synthesis and hormone signal transduction pathways, whereas DAMs were primarily enriched in ascorbate and aldarate metabolism, phenylpropanoid biosynthesis, and the pentose phosphate pathway. A combined multi-omics study showed that 116 DEGs and 3 DAMs in starch and sucrose metabolism (48 DEGs and 1 DAM), glycolysis and gluconeogenesis (54 DEGs and 1 DAM), and the pentose phosphate pathway (14 DEGs and 1 DAM) were significantly enriched. These findings suggest that blueberries predominantly increase sugar accumulation by activating carbon metabolism network pathways. Moreover, we identified critical transcription factors linked to the sugar response. This study presents new understandings regarding the molecular mechanisms underlying blueberry sugar accumulation and will be helpful in improving blueberry fruit quality through breeding.

## 1. Introduction

Blueberry, a plant of the genus *Vaccinium* in the Ericaceae, is an economic fruit tree with significant nutritional and health care value [1,2]. Blueberries are usually divided into four types, namely, lowbush blueberry, highbush blueberry, semi-highbush blueberry, and rabbiteye blueberry [3]. Blueberry fruits are oblate or spherical in shape and are dark blue or scarlet when ripe. Blueberry fruit is soft and juicier. It has an excellent level of antioxidant active ingredients, including polyphenols, flavonoids, anthocyanins, and ellagic acid, which have unique effects such as preventing vascular aging, protecting eyesight, anti-inflammation, anti-oxidation, and anti-cancer [1,2]. Blueberry fruit can be consumed fresh or processed into a number of items, including sauce, dried fruit, fruit juice, and fruit wine [4].

The manual cultivation of blueberries originated in North America [5]. In the past twenty years, blueberry cultivation and consumption have spread fast from North America to the rest of the world. Blueberry has been booming in China in recent years, and it is now readily available throughout the country. The farmed area is extensive in the Liaoning, Shandong, Jiangsu, and Yunnan provinces, resulting in significant economic benefits [6]. People in China are becoming more aware of the nutritional value of blueberries as the economy improves and consumer demand increases [7]. As a result, in order to better adapt to the needs of consumers, cultivating excellent blueberry varieties and improving the quality of blueberry fruits have become the focus of blueberry development and research in China [6,7].

Excellent fruit quality is a key factor in ensuring fruit commerciality and attracting consumers [8,9]. Fruit quality is defined primarily by the external appearance and internal quality of the fruit. The external quality is mostly determined by its size, hardness, and color, while the interior quality is determined by sugar, organic acids, aroma, and antioxidant active components [10,11]. Sugar is the main component of fruit quality and flavor, as well as the primary raw material for vitamin, aromatic compound, and pigment synthesis [11,12]. Recent studies have demonstrated that sugars can be employed as signaling molecules to participate in the metabolic processes of hormones, anthocyanins, nitrogen, and other substances [13,14,15]. As a result, in-depth research into the carbohydrate accumulation mechanism in the fruit is the foundation of the quality formation theory, and it is critical to increasing the quality of blueberry fruit.

The sugars in the mature fruits are mostly sucrose, fructose, and glucose [16,17]. Various investigations have discovered that starch accumulation and sugar metabolism in fruits are closely related to enzyme activity, but the mechanisms of sugar accumulation and the regulation of important enzymes vary with plant species [17]. Carbohydrate accumulation and metabolism have been studied in a variety of fruits, such as strawberry, citrus, pear, apricot, and pitaya [18,19,20,21,22]. Starch is the most important storage polysaccharide in plants [17]. The accumulation of starch maintains adequate carbon source reserves, while the decomposition products produced by amylase are utilized for sucrose synthesis [23]. Sucrose is the most commonly used form of assimilation product transit from “source” to “sink” in most plants [24]. Sucrose is a fruit quality component as well as a metabolic regulator and a signaling factor that either activates or inhibits the transcription of specific genes [24]. In plants, the enzymes closely related to sucrose metabolism include sucrose phosphate synthase (SPS), sucrose synthase (SUS), and invertase [25]. SPS is mainly responsible for sucrose biosynthesis; SUS catalyzes the reversible conversion of sucrose and fructose; and invertase is in charge of sucrose hydrolysis [20,22,26]. Wang et al. [26] discovered that increased SPS and SUS gene expression resulted in a considerable accumulation of sucrose in squash. There was a positive correlation between plum sucrose content and ChSPS1 activity [20]. Zhang et al. [22] reported that acid invertase was engaged in the regulation of fructose formation in pitaya through a transcriptome investigation.

Currently, research on carbohydrate metabolism in blueberry fruits mainly focuses on the variations in sugar content and related enzyme activity during fruit development and rarely involves the molecular mechanism [16,27]. Therefore, the understanding of the mechanism of carbohydrate accumulation in different cultivars of blueberry fruits is still in its infancy. Comprehensive analysis of transcriptomics and metabolomics is vital for understanding the complex regulatory network of carbohydrate metabolism in fruits. In recent years, multi-omics integration has been used to better understand the signaling pathways and mechanisms of sugar accumulation in fruits. Yang et al. [28] investigated the main causes of variations in sweet cherry quality at different developmental stages, including sugar, organic acids, and flavonoids, through the combination of transcriptome and metabolomics. Gao et al. [29] comprehensively analyzed the molecular mechanism of the variation in sugar accumulation at different developmental stages of the two pineapple cultivars through transcriptome and metabolomics. However, the mechanisms underlying sugar accumulation in blueberry fruits have not been well studied. Based on a comprehensive study of fruit quality, an association analysis of transcriptomics and metabolomics was performed to investigate the gene function and gene regulatory network associated with carbohydrate metabolism in different blueberry cultivars. In the present work, we explored the response mechanisms of different genes and metabolites in carbohydrate metabolism in different blueberry cultivars and created a carbohydrate metabolism regulation network. The findings will help to deepen the understanding of the molecular regulatory mechanisms of blueberry fruit sugar metabolism and facilitate the molecular breeding of high-sugar blueberry cultivars.

## 2. Results

### 2.1. Fruit Appearance, Antioxidant System, Bioactive Compounds and Flavor Indicators

**Figure 1:**
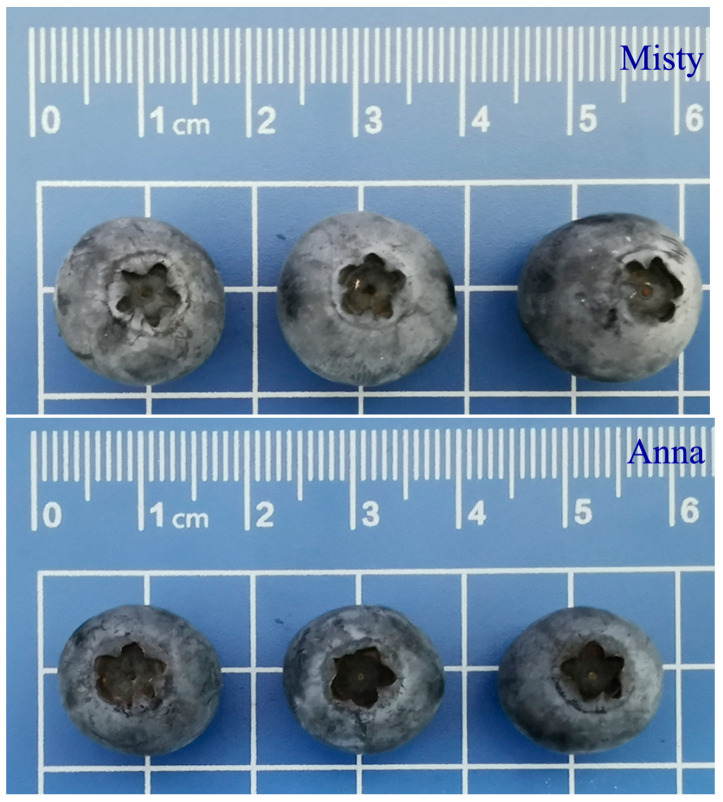
Fruit appearance of ‘Anna’ and ‘Misty’ blueberry cultivars.

**Figure 2:**
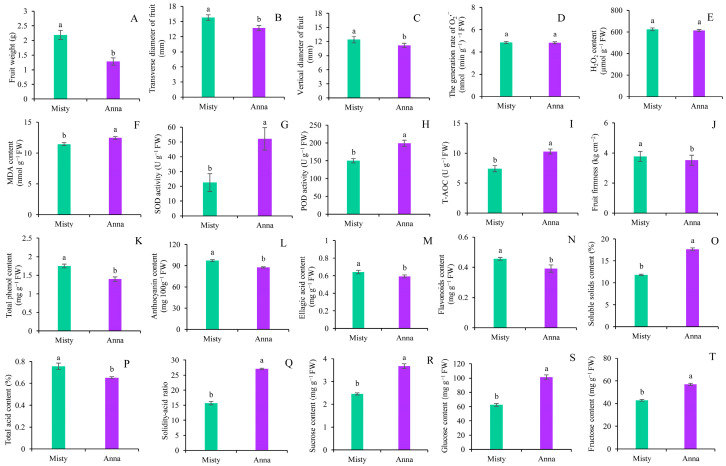
Antioxidant system, bioactive substances, and flavor indexes of ‘Anna’ and ‘Misty’ blueberry cultivars. (**A**) fruit weight, (**B**) transverse diameter, (**C**) vertical diameter, (**D**) the generation rate of O_2_^·−^, (**E**) H_2_O_2_ content, (**F**) MDA content, (**G**) SOD activity, (**H**) POD activity, (**I**) T-AOC, (**J**) fruit firmness, (**K**) total phenol content, (**L**) anthocyanin content, (**M**) ellagic acid content, (**N**) flavonoids content, (**O**) soluble solids content, (**P**) total acid content, (**Q**) solidity-acid ratio, (**R**) sucrose content, (**S**) glucose content, and (**T**) fructose content were measured in blueberry fruits. Columns with different letters indicate significant differences between ‘Anna’ and ‘Misty’ (*p* < 0.05).

Sugars are the primary components that influence the sweetness and taste of blueberry fruit. Blueberries vary in their sugar composition and quantity. In order to understand the accumulation mechanism of sugar substances in blueberry fruits, in this experiment, two blueberry cultivars, ‘Anna’ and ‘Misty’ (Figure 1), were selected, with obvious differences in sugar content, and the differences in appearance and quality of these two cultivars were systematically studied. In this study, the single fruit weight of ‘Misty’ was much greater than that of ‘Anna’, which was 71.2% heavier than that of ‘Anna’ (Figure 2A). Furthermore, the transverse diameter was 14.87% larger than ‘Anna’, while the vertical diameter was 10.96% larger (Figure 2B,C).

Excessive reactive oxygen species (ROS) production during ripening and senescence can cause oxidative damage to fruits. To maintain sufficient ROS homeostasis, plants produce a variety of free radical scavengers, primarily composed of antioxidant compounds or antioxidant enzymes. In the present study, the generation rate of O_2_^·−^ and H_2_O_2_ content in the fruits of the two cultivars did not differ dramatically. As compared to ‘Misty’ fruit, ‘Anna’ fruit had higher SOD and POD activities, as well as total antioxidant capacity (T-AOC), which could be attributed to its higher MDA level (Figure 2D–I).

Firmness is the main indicator used to assess fruit softening, and polyphenols, anthocyanins, flavonoids, and ellagic acid are the main active components of blueberry fruit. The study found that the hardness and the main active components concentrations of ‘Anna’ fruits were obviously lower than those of ‘Misty’ fruits, but the soluble solids content and solid-to-acid ratio of ‘Anna’ fruit were considerably greater, by 49.3% and 72.8%, respectively (Figure 2J–Q). In addition, ‘Anna’ fruit had a lower total acid content than ‘Misty’ fruit. The concentrations of sucrose, glucose, and fructose in ‘Anna’ fruit were considerably greater than those in ‘Misty’ fruit, which were 49.65%, 62.69%, and 33.16% higher, respectively (Figure 2R–T). These results indicated that the ‘Anna’ fruit has a sweet taste and good flavor, but the content of the functional components is slightly lower.

### 2.2. Transcriptomic Analysis

**Figure 3:**
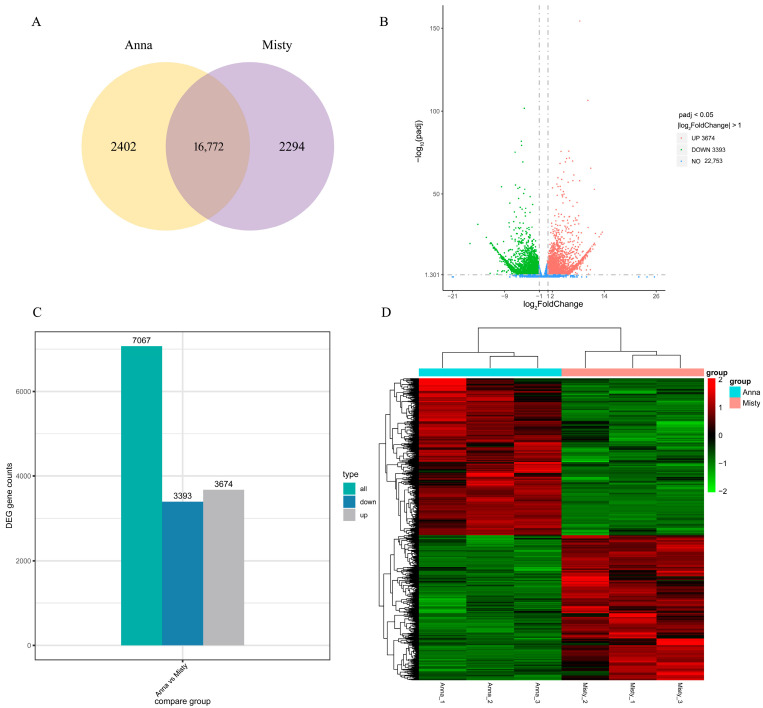
Transcriptomic analysis of the ‘Anna’ and ‘Misty’ blueberry cultivars. (**A**) Venn diagram of all identified genes. (**B**) Volcano plot of all identified genes; red represents up-regulated genes, green represents down-regulated genes, and blue represents genes that did not change significantly. (**C**) The number of DEGs. (**D**) Heatmap of all identified genes.

Transcriptome sequencing was used to investigate changes in gene expression among blueberry fruits of different cultivars. The sequencing data quality was high, with 42.9–46.1 million clean reads and 6.7–6.72 G clean bases acquired; Q30 was between 92.47 and 93.63%, and GC content was between 46.27 and 46.77%. The total mapped reads were greater than 81%, indicating that the sequencing data were sufficiently aligned for further analysis (Table S1). According to Figure 3A, the two samples co-expressed a total of 16,772 genes, with 2402 and 2294 genes expressed specifically in the ‘Anna’ and ‘Misty’ groups of samples, respectively.

With ‘Misty’ fruit as the control group and log_2_ (fold change) ≥ 1 and padj ≤ 0.05 genetic variations for the filter screen, 7067 differentially expressed genes (DEGs) were identified, of which 3674 DEGs were up-regulated and 3393 DEGs were down-regulated. They made up 51.99% and 48.01% of the total (Figure 3B,C). A hierarchical clustering analysis of DEGs indicated considerable variations between the two cultivars in gene expression patterns (Figure 3D). With strong repeatability, the three replicates of each group clustered into one class (Figure 3D).

### 2.3. GO and KEGG Enrichment Analysis

**Figure 4:**
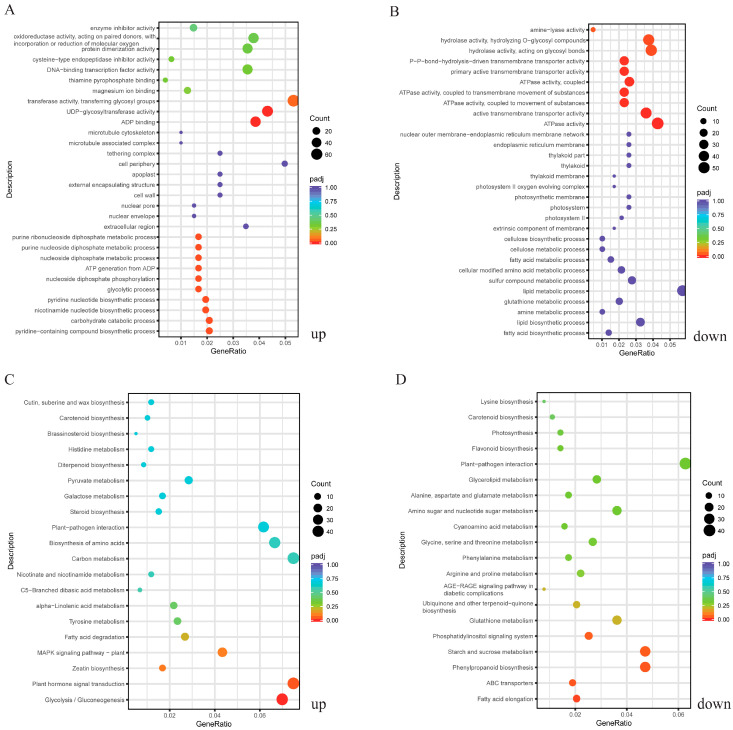
GO enrichment analysis of the up-regulated DEGs (**A**) and down-regulated DEGs (**B**). KEGG enrichment analysis of the up-regulated DEGs (**C**) and down-regulated DEGs (**D**).

As shown in Figure 4A, the top five significantly enriched GO terms among the up-regulated DEGs were ‘ADP binding’, ‘UDP-glycosyltransferase activity’, ‘pyridine-containing compound metabolic process’, ‘carbohydrate catabolic process’, and ‘nicotinamide nucleotide biosynthetic process’. Among the down-regulated DEGs, ‘ATPase activity’, ‘active transmembrane transporter activity’, ‘ATPase activity, coupled to transmembrane movement of substances’, ‘ATPase activity, coupled to movement of substances’, and ‘ATPase activity, coupled’ were the top five highly enriched GO terms (Figure 4B).

According to KEGG enrichment analysis, the top five significantly enriched pathways among the up-regulated DEGs were ‘glycolysis/gluconeogenesis’, ‘plant hormone signal transduction’, ‘zeatin biosynthesis’, ‘MAPK signaling pathway-plant’, and ‘fatty acid degradation’ (Figure 4C). Among the down-regulated DEGs, the top five significantly enriched pathways were ‘fatty acid elongation’, ‘ABC transporters’, ‘phenylpropanoid biosynthesis’, ‘starch and sucrose metabolism’, and ‘the phosphatidylinositol signaling system’ (Figure 4D).

### 2.4. DEGs in Phenylpropanoid Biosynthesis and Plant Hormone Signal Transduction Pathways

The phenylpropanoid biosynthesis pathway is a major source of phenolic compound accumulation in plants. Based on the KEGG enrichment analysis, this pathway was considerably enriched, and 45 genes were differentially expressed, with 15 genes significantly elevated and 30 genes greatly down-regulated (Figure S1). Among them, two 4-coumarate-CoA ligase (4CL) genes, four beta-glucosidase (BG1) genes, one caffeate O-methyltransferase (COMT) gene, one shikimate o-hydroxycinnamoyltransferase (HCT) gene, one cytochrome P450 monooxygenase 98 (CYP98) gene, four peroxidase (POD) genes, and two cinnamyl-alcohol dehydrogenase (CAD) genes were highly up-regulated. Hormone signaling is closely linked to sugar metabolism. In the plant hormone signal transduction pathway, 70 DEGs were identified (45 up-regulated and 25 down-regulated). The auxin and brassinosteroid hormone signal transduction pathways were highly enriched in 21 (14 up-regulated and 7 down-regulated) and 17 (11 up-regulated and 6 down-regulated) DEGs, respectively (Figure S2). In the auxin signal transduction pathway, one auxin influx carrier (AUX1) gene, four auxin-responsive protein IAA (AUX/IAA) genes, one auxin response factor (ARF) gene, two auxin-responsive GH3 gene family (GH3) genes, and six SAUR family protein (SAUR) genes were highly up-regulated. In the brassinosteroid signal transduction pathway, four brassinosteroid insensitive 1-associated receptor kinase 1 (BAK1) genes, one protein brassinosteroid insensitive 1 (BRI) gene, one protein brassinosteroid insensitive 2 (BIN1) gene, four xyloglucosyl transferase (TCH4) genes, and one cyclin D3 (CYCD3) gene were considerably up-regulated. These up-regulated genes may play a major role in sugar metabolism.

### 2.5. Transcription Factors (TFs)

**Figure 5:**
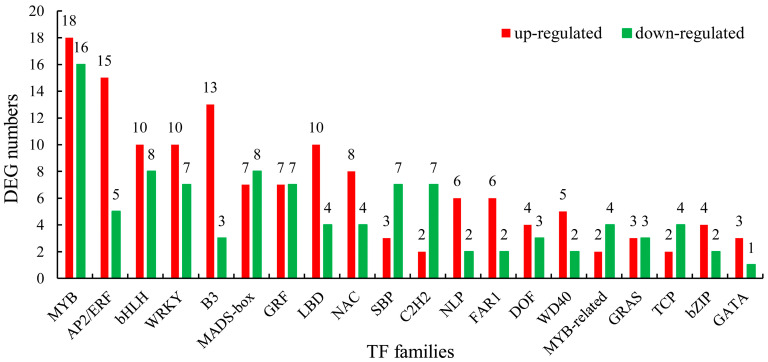
Number of DEGs involved in the top 20 TF families.

Transcription factors (TFs) can either promote or suppress target gene transcription. To further understand the differential expression of TFs between the fruits of these two cultivars, TF families with a DEG count in the top 20 were identified (Table S2). As shown in Figure 5, the MYB family had the most DEGs (34 genes), with 18 of them being up-regulated and 16 being down-regulated. The number of DEGs that were up-regulated outnumbered those that were down-regulated in 13 transcription factor families. There were more than 15 DEGs in numerous transcription factor families, including MYB, AP2/ERF, bHLH, WRKY, B3, and MDAS-box. Within these six TF families, there were 18, 15, 10, 10, 13, and 7 significantly up-regulated genes, respectively. Among these up-regulated genes, a total of ten genes had log_2_ (fold change) values greater than six, including two MYB genes (gene-Vadar_022042 and gene-Vadar_028064), one AP2/ERF gene (gene-Vadar_012138), three bHLH genes (gene-Vadar_024786, gene-Vadar_021192, and gene-Vadar_000396), one WRKY gene (gene-Vadar_014305), two B3 genes (novel.480 and gene-Vadar_002518), and one MADS-box gene (gene-Vadar_003970) (Table S3).

### 2.6. Metabolic Profiling

**Figure 6:**
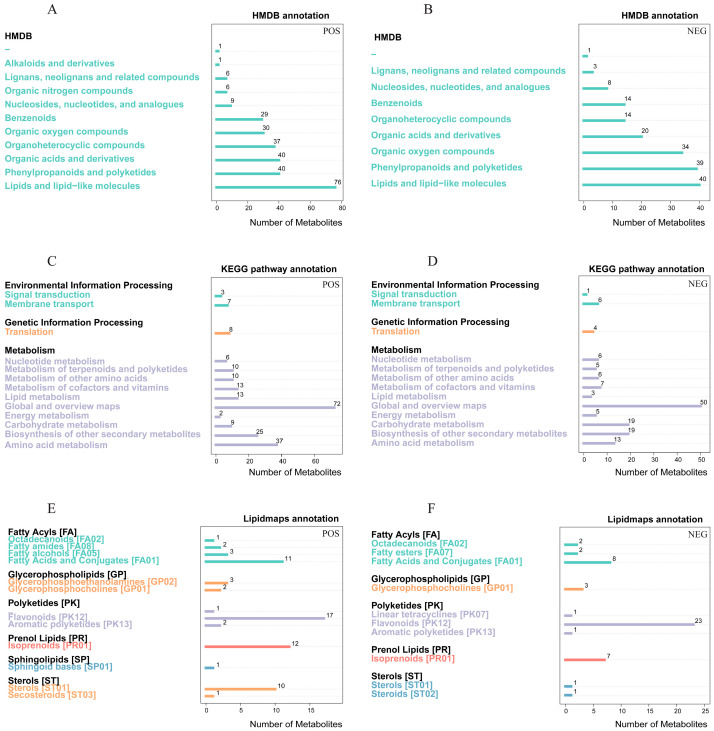
Metabolite profiling and classification using the HMDB, KEGG, and LIPID MAPS databases. The HMDB annotation of the metabolites in the positive (**A**) and negative (**B**) ion modes. The KEGG pathway annotation of the metabolites in the positive (**C**) and negative (**D**) ion modes. The LIPID MAPS annotation of the metabolites in the positive (**E**) and negative (**F**) ion modes.

In all, 845 metabolites were found in blueberry fruits, with 536 detected in the positive ion mode and 309 found in the negative ion mode (Table S4). These metabolites were further annotated and functionally categorized employing the HMDB, KEGG, and LIPID MAPS databases (Figure 6). According to the HMDB annotation, the metabolites were mostly lipids and lipid-like molecules, organic acids and derivatives, phenylpropanoids and polyketides, organoheterocyclic compounds, organic oxygen compounds, and benzenoids. The KEGG pathway annotation found that in the positive ion mode, the metabolites were primarily abundant in amino acid metabolism, biosynthesis of other secondary metabolites, and lipid metabolism pathways. In the negative ion mode, the metabolites were mainly enriched in the biosynthesis of other secondary metabolites, carbohydrate metabolism, and amino acid metabolism. The annotation of LIPID MAPS indicated that the metabolites were primarily flavonoids, isoprenoids, and fatty acids and conjugates.

### 2.7. Metabolite Multivariate Statistical Analysis

Principal component analysis (PCA) was used on the two sample groups to differentiate between changes in metabolite accumulation in the fruits. As shown in Figure S3A,B, the PC1 can explain 35.62% and 38.16% of the total variation in the positive and negative ion modes, respectively, and the PC2 can explain 26.89% and 27.83% of the total variance, respectively. There is a clear differentiation between the samples (Figure S3). PLS-DA score plot analysis showed that the two samples were clearly separated and clustered inside the group (Figure S3C,D). In the positive and negative ion modes, the R2Y (cum) values of the PLS-DA model were 0.99 and 1, respectively, demonstrating that the model could effectively display metabolite differences between the sample groups. Furthermore, 200 response permutation tests were used to validate the accuracy of the PLS-DA model, and the findings revealed that the constructed model was stable and dependable, and that it could be used for subsequent differential metabolite screening (Figure S3E,F).

### 2.8. DAM Screening and Analysis

In this study, VIP > 1.0, FC > 1.5, or FC < 0.667 and *p*-value < 0.05 were used as screening criteria to screen DAMs. In the positive ion mode, 70 DAMs were screened, 44 of which were up-regulated and 26 of which were down-regulated. A total of 70 DAMs were screened in the negative ion mode, with 38 DAMs up-regulated and 32 DAMs down-regulated (Figure S4A,B). The results of the hierarchical clustering analysis showed that there was a substantial difference between the two sample groups and that the DAMs within the same group were clustered together. The similarity within groups was high, and these DAMs could be used for group difference analysis. Correlation analysis of the top 20 DAMs showed that, with the exception of di-C,C-pentosyl-apigenin, betulin, 4-oxododecanedioic acid, Bruceine D, stigmasterol, and combretastatin A4, most DAMs were strongly positively associated with each other in the positive ion mode. N6-isopentenyladenosine was found to be positively associated to 4-chloro-5-morpholino-2-quinoxalin-2-ylpyridazin-3(2H)-one and N-[(4-hydroxy-3-methoxyphenyl)methyl]-8-methylnonanamide. In the negative ion mode, ixoside, geniposidic acid, benzyl 6-O-beta-D-glucopyranosyl-beta-D-glucopyranoside, and 8-epiloganic acid were positively correlated with each other. Stevioside was only positively correlated with hydroxysafflor yellow A (Figure S4C,D).

### 2.9. KEGG Enrichment Analysis of DAMs

**Figure 7:**
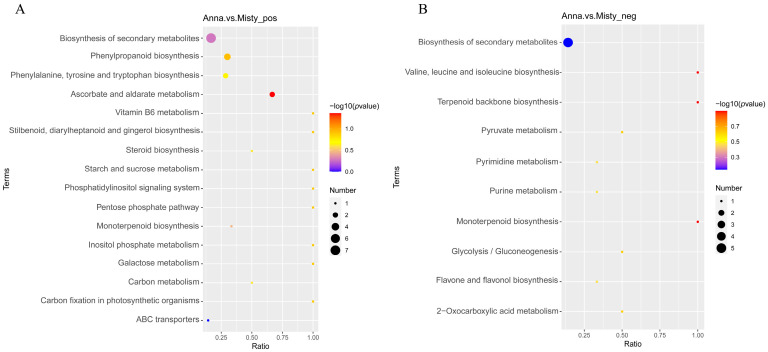
KEGG enrichment analysis of DAMs in the positive (**A**) and negative (**B**) ion modes.

Pathways hosting DAMs in blueberry fruits were analyzed by the KEGG database. The study found that DAMs were primarily enriched in 17 metabolic pathways in the positive ion mode, including ascorbate and aldarate metabolism, phenylpropanoid biosynthesis, the pentose phosphate pathway, galactose metabolism, and starch and sucrose metabolism, which were the top five significantly enriched metabolic pathways (Figure 7A). The DAMs were primarily enriched in 10 metabolic pathways in the negative ion mode, including valine, leucine, and isoleucine biosynthesis, terpenoid backbone biosynthesis, monoterpenoid biosynthesis, glycolysis/gluconeogenesis, and pyruvate metabolism, which were the top five significantly enriched metabolic pathways (Figure 7B).

### 2.10. Transcriptomics and Metabolomics Correlation Analysis

**Figure 8:**
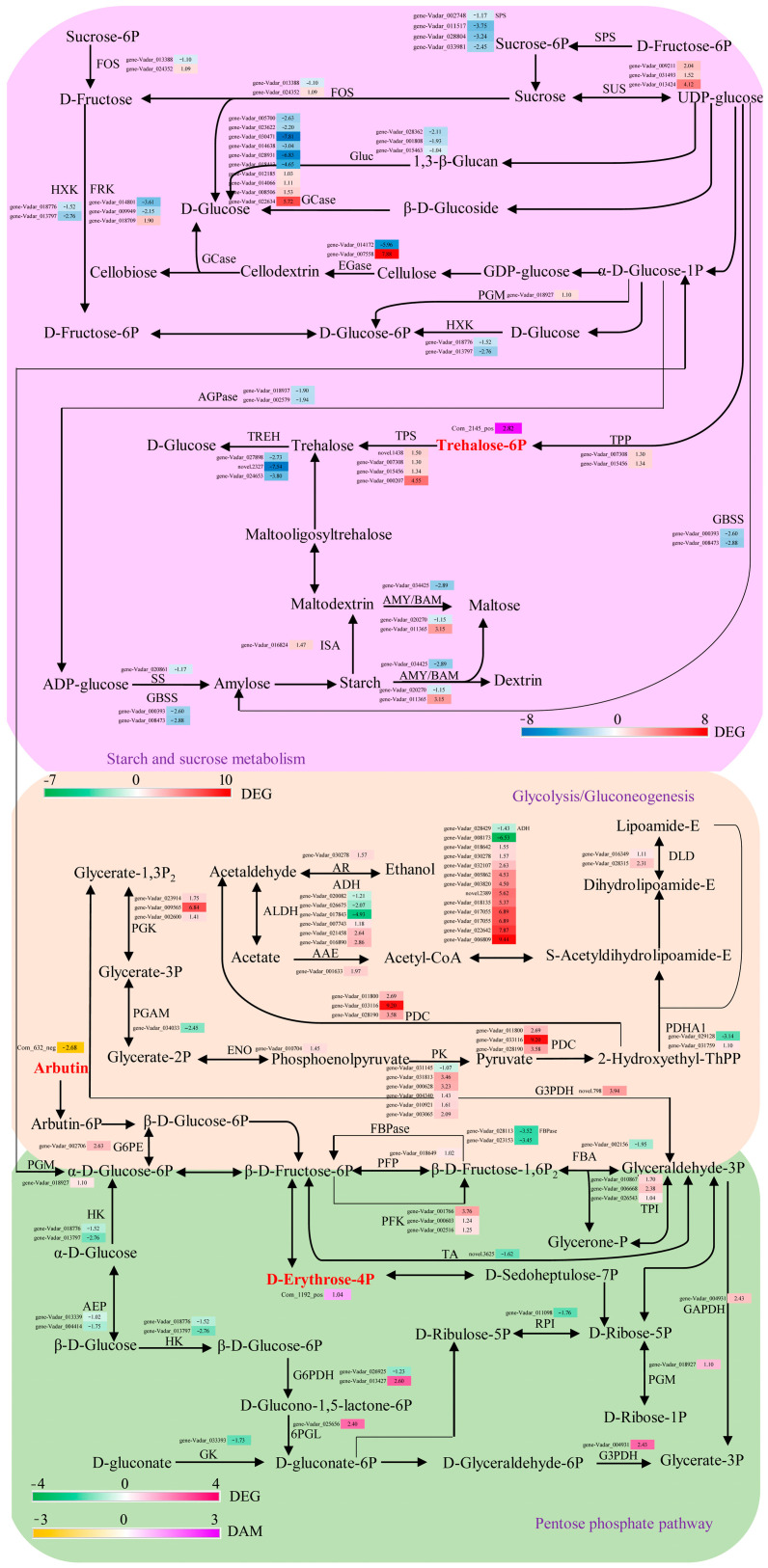
DAMs and DEGs association studies in starch and sucrose metabolism, glycolysis/gluconeogenesis, and the pentose phosphate pathway. The scales were generated using log_2_ (fold change) values. The expression levels of DEGs and DAMs are shown from green to red and yellow to purple (low to high) in the comparison of ‘Anna’ vs. ‘Misty’, respectively. HXK, hexokinase; FRK, fructokinase; FOS, beta-fructofuranosidase; Gluc, glucan endo-1,3-beta-glucosidase; GCase, beta-glucosidase; EGase, endoglucanase; SPS, sucrose-phosphate synthase; SUS, sucrose synthase; PGM, phosphoglucomutase; AGPase, ADP-glucose pyrophosphorylase; TPS, trehalose 6-phosphate synthase; TPP, trehalose 6-phosphate phosphatase; TREH, alpha,alpha-trehalase; GBSS, granule-bound starch synthase; AMY, alpha-amylase; BAM, beta-amylase; ISA, isoamylase; SS, starch synthase; HK, hexokinase; AEP, aldose 1-epimerase; G6PE, glucose-6-phosphate 1-epimerase; FBPase, fructose-1,6-bisphosphatase; PFK, ATP-dependent phosphofructokinase; PFP, pyrophosphate-dependent fructose-6-phosphate phosphotransferase; FBA, fructose-bisphosphate aldolase; TPI, triosephosphate isomerase; G3PDH, glyceraldehyde 3-phosphate dehydrogenase; GAPDH, glyceraldehyde-3-phosphate dehydrogenase; PGK, phosphoglycerate kinase; PGAM, 2,3-bisphosphoglycerate-dependent phosphoglycerate mutase; ENO, enolase; PK, pyruvate kinase; PDHA1, pyruvate dehydrogenase E1 component subunit alpha; PDC, pyruvate decarboxylase; DLD, dihydrolipoyl dehydrogenase; AAE, butyrate-CoA ligase; ALDH, aldehyde dehydrogenase; ADH, alcohol dehydrogenase; AR, aldose reductase; GK, gluconokinase; G6PDH, glucose-6-phosphate 1-dehydrogenase; 6PGL, 6-phosphogluconolactonase; TA, transaldolase; RPI, ribose 5-phosphate isomerase A.

In order to gain a deeper comprehension of the differences in carbohydrate accumulation between the two blueberry cultivars, we performed a combined transcriptome and metabolomic analysis on three pathways (starch and sucrose metabolism, glycolysis/gluconeogenesis, and pentose phosphate pathway) in the carbon metabolism network (Figure 8).

The study found that, in the starch and sucrose metabolism pathway, there were 48 DEGs, i.e., two hexokinase (HXK) genes, three fructokinase (FRK) genes, two beta-fructofuranosidase (FOS) genes, three glucan endo-1,3-beta-glucosidase (Gluc) genes, ten beta-glucosidase (GCase) genes, two endoglucanase (EGase) genes, four sucrose-phosphate synthase (SPS) genes, three sucrose synthase (SUS) genes, one phosphoglucomutase (PGM) gene, two ADP-glucose pyrophosphorylase (AGPase) genes, two trehalose 6-phosphate phosphatase (TPP) genes, four trehalose 6-phosphate synthase (TPS) genes, three alpha-trehalase (TREH) genes, two granule-bound starch synthase (GBSS) genes, one alpha-amylase (AMY) gene, two beta-amylase (BAM) genes, one isoamylase (ISA) gene, and one starch synthase (SS) gene and one DAM (Trehalose 6-phosphate (Com_2145_pos)) were significantly enriched. In this pathway, 21 DEGs were substantially up-regulated.

In the glycolysis/gluconeogenesis pathway, 54 DEGs, i.e., one PGM gene, two hexokinase (HK) genes, two aldose 1-epimerase (AEP) genes, one glucose-6-phosphate 1-epimerase (G6PE) gene, two fructose-1,6-bisphosphatase (FBPase) genes, three 6-phosphofructokinase (PFK) genes, one diphosphate-dependent phosphofructokinase (PFP) gene, one fructose-bisphosphate aldolase (FBA) gene, three triosephosphate isomerase (TPI) genes, one glyceraldehyde 3-phosphate dehydrogenase (G3PDH) gene, one glyceraldehyde-3-phosphate dehydrogenase (GAPDH) gene, one phosphoglycerate kinase (PGK) gene, one 2,3-bisphosphoglycerate-dependent phosphoglycerate mutase (PGAM) gene, one enolase (ENO) gene, six pyruvate kinase (PK) genes, two pyruvate dehydrogenase E1 component subunit alpha (PDHA1) genes, three pyruvate decarboxylase (PDC) genes, two dihydrolipoyl dehydrogenase (DLD) genes, one acetate/butyrate-CoA ligase (AAE) gene, six aldehyde dehydrogenase (ALDH) genes, twelve alcohol dehydrogenase (ADH) genes, and one aldose reductase (AR) gene, and one DAM (arbutin (Com_632_neg)) showed synergistic changes. The majority of the DEGs in this pathway, particularly the ADH genes, were considerably up-regulated.

In the pentose phosphate pathway, 14 DEGs, i.e., one gluconokinase (GK) gene, two glucose-6-phosphate 1-dehydrogenase (G6PDH) genes, one 6-phosphogluconolactonase (6PGL) gene, one GAPDH gene, two FBPase genes, one PFP gene, three PFK genes, one transaldolase (TA) gene, one PGM gene, and one ribose 5-phosphate isomerase A (RPI) gene and one DAM (D-erythrose 4-phosphate (Com_1192_pos)) demonstrated synergistic alterations.

Among the DEGs in these three pathways, six had log_2_ (fold change) values larger than six, including one EGase gene (gene-Vadar_007558), one PGK gene (gene-Vadar_009565), one PDC gene (gene-Vadar_033116), and three ADH genes (gene-Vadar_017055, gene-Vadar_022642, and gene-Vadar_006809). These genes may be crucial for fruit sugar metabolism (Table S3). Furthermore, to corroborate the RNA-seq data, 12 DEGs from the starch and sucrose metabolism pathway and 12 DEGs from the glycolysis/gluconeogenesis pathway were selected for qRT-PCR analysis. The results of qRT-PCR were consistent with those of transcriptomics (Figure S5), indicating the high degree of accuracy of the transcriptome sequencing results.

## 3. Discussion

Blueberry quality has become increasingly important as people’s living levels have improved in recent years [11]. Improving blueberry fruit quality has emerged as a key research avenue for adapting to market development and an essential objective of modern agricultural blueberry production [9,10]. The purpose of this study was to gain insight into the changes in genes and metabolites in ripe fruits of the high-sugar blueberry cultivar ‘Anna’ and the common blueberry cultivar ‘Misty’, as well as their link to fruit quality. This research might be used to choose molecular genetic targets for enhancing carbohydrate accumulation in blueberries.

Blueberry fruit quality refers to the total of several positive features that suit people’s needs [10]. In this study, it was found that the size and firmness of the ‘Anna’ fruit were significantly lower than those of the ‘Misty’ fruit. Active compounds, such as polyphenols, anthocyanins, ellagic acid, and flavonoids, were substantially lower in ‘Anna’ fruit than in ‘Misty’ fruit. The appearance of fruits is an essential aspect of attracting customers to buy them [30]. Fruits with beautiful shapes have a higher market value, and most consumers prefer large fruit varieties. The hardness of fruits influences not only their storage properties but also their taste [31]. Antioxidant active substances with various health care functions are also important indicators of blueberry quality [1,2]. According to this study, the commercial performance of ‘Anna’ fruit was much lower than that of ‘Misty’ fruit, which limited the marketing and cultivation of this cultivar in China. The analysis of the expression levels of the genes involved in the phenylpropanoid biosynthesis pathway revealed that 45 genes were variously expressed, and the expression levels of the majority of genes were drastically reduced. The study concluded that the ability of ‘Anna’ fruit to synthesize secondary metabolites was significantly lower than that of ‘Misty’ fruit, which was the main reason for the lower content of active substances in this cultivar. In addition, the MDA content, SOD activity, POD activity, and T-AOC value of ‘Anna’ fruit were much higher than those of ‘Misty’ fruit. The higher antioxidant capability may be used to repair membrane lipid peroxidation damage and maintain the equilibrium of reactive oxygen species (ROS) metabolism inside the fruit [11]. This could be related to the lower levels of polyphenols, anthocyanins, ellagic acid, and flavonoids in ‘Anna’ fruit, as these active components can help plants resist free radical stress.

The sugar content and composition in fruits are primary indicators of fruit flavor [16]. Sugars found in ripe fruits are primarily sucrose, fructose, and glucose [22,26]. According to recent research, ripening ‘O’Neal’ blueberry fruits primarily accumulate fructose and glucose, followed by sucrose [27]. Similarly, soluble solids, sugar-acid ratio, fructose, glucose, and sucrose contents of ‘Anna’ fruits were found to be much higher than those of ‘Misty’ fruits, but titratable acid content was significantly lower. Sugar accumulation is caused not only by the “sink/source” relationship between photosynthetic organs and fruits [24], but also by the respiratory metabolism process, which promotes the improvement of fruit quality, the transformation of flavor substances, and the presentation of ripening quality [32,33,34,35].

To explore the variations in sugar accumulation in blueberry fruits, this study used combined transcriptome and metabolome analysis to identify three key metabolic pathways related to carbon metabolism: starch and sucrose metabolism, glycolysis/gluconeogenesis, and the pentose phosphate pathway. The metabolism of starch and sucrose is a complicated biochemical process that relies on the synergistic action of multiple enzymes [17,23,24]. In the starch and sucrose metabolism pathway, 48 DEGs and one DAM (trehalose 6-phosphate) were discovered. SPS is a crucial enzyme in sucrose production, whereas SUS is involved in reversible sucrose metabolism and primarily performs a catabolic role [20,26]. As compared to ‘Misty’ fruit, the expression of four SPS genes was considerably decreased, and three SUS genes were greatly promoted in ‘Anna’ fruit, which could promote sucrose breakdown and increase fructose and glucose levels. In plants, SS and GBSS are responsible for starch synthesis, while AMY and BAM are the main players in starch degradation [23]. Significant down-regulation of SS (gene-Vadar_020861) and GBSS (gene-Vadar_000393, gene-Vadar_008473) genes inhibited starch synthesis. The up-regulation of the BAM (gene-Vadar_011365) gene further promoted starch degradation. The down-regulation of AGPase (gene-Vadar_018937, gene-Vadar_002579) genes reduced the production of ADP-glucose, a substrate for starch synthesis [17]. In addition, the trehalose 6-phosphate (T6P) content of the ‘Anna’ fruit was substantially higher than that of the ‘Misty’ fruit. The considerable overexpression of the TPP genes (gene-Vadar_007308, gene-Vadar_015456) should be related to this. T6P functions as a signal substance for the sufficiency of carbon sources [36], as well as delaying leaf senescence and influencing fruit setting rate [37,38]. Therefore, its function in ‘Anna’ fruit deserves further investigation.

Sugar metabolism and respiratory metabolism in fruits are tightly connected. Fructose 6-phosphate (F6p), produced by starch and sucrose metabolism, enters the glycolysis/gluconeogenesis and the pentose phosphate pathway to produce energy and serves as an intermediary in other metabolic processes to ensure fruit development [39,40]. The study identified 54 and 14 DEGs and two DAMs (arbutin and D-erythrose 4-phosphate) from glycolysis/gluconeogenesis and the pentose phosphate pathway, respectively. The glycolysis/gluconeogenesis pathway is a set of processes that convert glucose to pyruvate while producing ATP [39]. Phosphofructokinase and pyruvate kinase (PK) are two key enzymes in this pathway. Phosphofructokinase catalyzes the interconversion of F6P to fructose-1,6-diphosphate (FDP). There are two kinds of phosphofructokinases in plants: diphosphate-dependent phosphofructokinase (PFP) and ATP-dependent phosphofructokinase (PFK) [40]. Transient *PbPFP1* gene transformation in pear fruit resulted in a considerable rise in fructose and sorbitol [35]. MdMYB108 in apples could bind to the MdPFK promoter and induce soluble sugar accumulation in the pulp [41]. PK is in charge of converting the high-energy phosphate group of phosphoenolpyruvate (PEP) to ADP in order to create ATP and pyruvate [42]. By modulating cellular metabolic flux and ATP synthesis, PK regulates several aspects of plant growth and development [34,43]. Qin et al. [43] found that the expression level of *EjPK* in high-sugar loquat varieties was significantly higher than that in low-sugar loquat varieties, suggesting its potential role in the regulation of sugar content in loquat fruits. Guan et al. [34] demonstrated that PK was involved in the natural deastringence process of persimmon at the late stage of ripening. In this study, one PFP, three PFK, and five PK genes were considerably up-regulated, indicating that ‘Anna’ fruit has a high glycolysis rate. The increased sugar content of ‘Anna’ fruit may be due to the differential expression of PFP, PFK, and PK genes.

In addition, one PDC gene (gene-Vadar_033116) and three ADH genes (gene-Vadar_017055, gene-Vadar_022642, and gene-Vadar_006809) were found to be significantly up-regulated between the two blueberry cultivars. PDC catalyzes pyruvate decarboxylation to acetaldehyde, whereas ADH mainly promotes acetaldehyde and ethanol interconversion [44]. ‘Anna’ fruits produce tiny amounts of ATP via the ethanol fermentation pathway, which converts NAD(P)H to NAD(P) and maintains the metabolic balance of the glycolysis/gluconeogenesis pathway [45,46]. Alcohols are one of the limiting factors for ester production, and previous research revealed that ADH is also involved in the ripening of plant fruits. ADH activity increased in the late stages of tomato and melon fruit development, regulating alcohol production and providing fruits a more mature flavor [32,33]. The upregulation of ADH genes may also contribute to the flavor development of ‘Anna’ fruit.

The primary physiological functions of the pentose phosphate pathway are to produce NADPH for reducing biosynthesis, pentose phosphate for nucleic acid metabolism, and some intermediates involved in amino acid and fatty acid synthesis [47,48,49]. Glucose-6-phosphate dehydrogenase (G6PDH) is a key rate-limiting enzyme that catalyzes the dehydrogenation of glucose-6-phosphate to gluconic acid 6-phosphate and NADPH [47]. The considerable upregulation of G6PDH (gene Varadar_013427) in our study may further increase NADPH synthesis in ‘Anna’ fruit. NADPH also functions as a cofactor of glutathione reductase, ensuring enough reduced glutathione content [49]. Because of the low quantity of active compounds, such as polyphenols and anthocyanins, in ‘Anna’ fruit, NADPH may have a key role in modulating oxidative stress in the fruit. Furthermore, the significantly enhanced D-erythrose 4-phosphate may stimulate fructose-6P synthesis, accelerating glycolysis and increasing sugar accumulation in ‘Anna’ fruits.

Sugar is not only an energy source and an important component of plant structural substances; it also acts as a signal molecule that regulates various metabolic activities in plants in conjunction with signals such as hormones and nitrogen, and it participates in the expression of related genes via signal transduction [13,15,50]. According to studies, auxin and BR are hormones with obvious links to sugar sensing and signaling. Glucose regulates the expression of genes responsible for auxin synthesis, auxin transport, and auxin signal transduction [51,52]. Sugar modulates *Arabidopsis* hypocotyl elongation along the BR signaling pathway [53]. Hao et al. [53] found that the *LecRK-VIII.2* gene regulated hypocotyl elongation by integrating the signaling cross-talk among BR, sucrose, glucose, and fructose in *Arabidopsis*. This gene played a pivotal role in mediating BZR1 target genes, which were co-regulated by BR and sugar. BR has a significant effect on sugar accumulation in fruits and increasing endogenous BR levels greatly increased sugar accumulation in grape and citrus fruits [54]. In this study, sugars in ‘Anna’ fruit were hypothesized to be critical signaling molecules that regulate auxin and brassinosteroid signaling. In tomato, *SlARF10* regulates fruit chlorophyll and sugar accumulation via regulating the transcriptional upregulation of *AGPase*, *SlGLK1*, *POR*, *CBP1*, and *CBP2* [55]. In Arabidopsis, BRI1 and BAK1 function genetically with G-protein subunits to regulate sugar-responsive growth and development [56]. In this study, there were 21 and 17 DEGs that were significantly enriched in the auxin and the brassinosteroid signal transduction pathways, respectively. One ARF gene (gene-Vadar_000924) was highly up-regulated in the auxin signal transduction pathway. Four BAK1 genes (gene-Vadar_023654, gene-Vadar_031910, gene-Vadar_012080, and gene-Vadar_028865) and one BRI gene (gene-Vadar_021721) were considerably up-regulated in the brassinosteroid signal transduction pathway. These genes were considered to be able to promote sugar accumulation in ‘Anna’ fruit. 

Sugar synthesis, transport, and distribution occur throughout the plant growth and development process. The complicated sugar metabolism and transduction regulatory network integrates various internal and external factors to maintain the growth and development process [17,57,58,59,60,61]. A variety of transcription factors have been implicated in sugar response process regulation. *MdMYB305* has been shown to increase the sugar content of red-fleshed apple fruit by modulating the expression of the sugar-related genes *MdCWI1*, *MdVGT3*, and *MdTMT2* [57]. *CitERF16* positively regulates the expression of *CitSWEET11d*, thereby facilitating sucrose accumulation in citrus fruit [58]. *MdbHLH3* promotes soluble sugar accumulation in apples by activating *MdPFP* expression [59]. *HpWRKY3* is linked to sugar accumulation in pitaya fruit by promoting the expression of *HpINV2* and *HpSuS1* [60]. Co-transformation of *MuMADS1* and *MaOFP1* in tomato ovate mutants results in a considerable rise in sugar content [61]. In this study, the transcription factor families with DEG counts in the top 20 were identified. Among the transcription factor families MYB, AP2/ERF, bHLH, WRKY, and MDAS-box, there were 18, 15, 10, 10, and 7 significantly up-regulated genes, respectively. Among them, two MYB genes (gene-Vadar_022042 and gene-Vadar_028064), one AP2/ERF gene (gene-Vadar_012138), three bHLH genes (gene-Vadar_024786, gene-Vadar_021192, and gene-Vadar_000396), one WRKY gene (gene-Vadar_014305), and one MADS-box gene (gene-Vadar_003970), which had log_2_ (fold change) values greater than six, may be closely related to the sugar response of blueberry fruits. These TFs may interact with genes involved in sugar metabolism, regulating the accumulation of sugars in blueberry fruits.

## 4. Materials and Methods

### 4.1. Plant Materials

The fruits of 4-year-old blueberry ‘Anna’ and ‘Misty’ cultivars were used as experimental materials, which were collected from Lishui Baima Industrial Park (119°11′ E, 31°36′ N), Nanjing, Jiangsu Province, China. The cultivation and management conditions of the chosen plants were consistent. After the fruits had fully matured, three trees from each cultivar were chosen, and fruits without any signs of disease were collected randomly. The fruits were snap-frozen in liquid nitrogen and transported to the laboratory, where they were maintained in a refrigerator at −80 °C and used for physiological and biochemical assessments, as well as transcriptome and metabolomic analysis.

### 4.2. Fruit Appearance Indicators and Firmness 

The fruit weight was assayed with an electronic balance. A digital Vernier caliper and a fruit firmness meter (Catalog No. 9300 (KM-5), Kyoto, Japan) were used to determine the fruit’s transverse and longitudinal diameters and firmness, respectively. 

### 4.3. Antioxidant-Related Parameters

The hydroxylamine chloride protocol was applied to determine the generation rate of O_2_^·−^ [62]. The concentration of malondialdehyde (MDA) in fruit was determined by monitoring thiobarbituric acid reactive compounds [63]. Hydrogen peroxide (H_2_O_2_) and total antioxidant capacity (T-AOC) testing kits were used to determine the H_2_O_2_ level and T-AOC (Nanjing Jiancheng Bioengineering Institute, Nanjing, China). Fresh fruit (1 g) was homogenized in 10 mL of filtered water and centrifuged at 8000 rpm for 10 min at 4 °C. The supernatant was used to determine the H_2_O_2_ concentration. First, 0.1 mL of supernatant was added to 1 mL of titanium sulfate solution. After reaction at 37 °C for 1 min, 1 mL of the ammonia was added to the mixture. The absorbance was measured at 405 nm. Fresh fruit (1 g) was ground on ice with 10 mL of 0.01 M phosphate buffer (pH 7.4) and then centrifuged at 8000 rpm for 10 min at 4 °C. The T-AOC was determined using the supernatant. A quantity of 0.1 mL of the extract was incubated in the water at 37 °C for 30 min with 1 mL of 0.01 M phosphate buffer (pH 7.4), 2 mL of chromogenic agent, and 0.5 mL of ferric salt solution. Then 0.2 mL of stop solution and 0.2 mL of clarificant were added to the mixture. After standing at room temperature for 10 min, the absorption value of the reaction solution was measured at 520 nm. The activity of superoxide dismutase (SOD) was analyzed using the nitro blue tetrazolium (NBT) technique [64]. The activity of peroxidase (POD) was assessed by determining the guaiacol oxidation rate at 470 nm [65].

### 4.4. Fruit-Quality-Related Parameters

The content of total phenol and anthocyanin was measured using the Folin-Ciocalteu [66] and the pH differential methods [67], respectively. A digital refractometer (Atago, WYT-A, Tokyo, Japan) was used to determine the soluble solid content. The content of total acid was assessed using potentiometric titration in accordance with the state standard GB/T12456-2008, with citric acid serving as a standard. Then the ratio of the soluble solid to the total acid was determined.

The state standard GB/T 20574-2006 was used to measure the total flavonoid content. First, the fresh fruit sample (3 g) was extracted for 45 min at 65 °C in 30 mL of 95% ethanol. Then, the mixture was centrifuged for 5 min at 5000 rpm at 4 °C. The reaction mixture contained 3 mL of the supernatant, 1 mL of 100 g L^−1^ Al(NO)_3_, 10 mL of 95% ethanol, and 1 mL of 9.8 g L^−1^ CH_3_COOK, and was diluted to 50 mL with deionized water. The absorbance was determined at 415 nm after 1 h of incubation at room temperature.

The ellagic acid content was measured using the method presented by Maas et al. [68]. The samples were extracted in 40% ethanol by ultrasonic shaking in a water bath for 20 min at 80 °C. The resultant solution was then centrifuged at room temperature for 10 min at 7000 rpm. The reaction mixture contained 1 mL of the supernatant and 4 mL of 0.1 M NaOH. The absorbance was obtained at 357 nm.

### 4.5. Transcriptomic Analysis

Total RNA was extracted from blueberry fruits with the Tengen polysaccharide polyphenol kit (QIAGEN, Frankfurt, Germany). To check whether the RNA samples extracted in this test met the requirements, a Nano Drop1000 spectrophotometer and an Agilent 2100 Bioanalyzer were used for analysis. After qualified detection, the library was constructed using a NEBNext^®^ Ultra™ Directional RNA Library Prep Kit for Illumina^®^. The main steps were performed by referring to the specifications.

Sequencing was performed on the platform of an illumina NovaSeq 6000 (illumina, San Diego, CA, USA). The original sequences after high-throughput sequencing were filtered and screened to obtain clean reads that could be used for subsequent analysis. Sequence comparison was conducted between clean reads and the blueberry reference genome (*Vaccinium darrowii*), using HISAT2 v2.0.5 [69]. Differentially expressed genes (DEGs) were screened under the conditions of |log_2_ (fold change)| ≥ 1 and padj ≤ 0.05 [70]. Functional annotation and classification of DEGs were carried out with reference to the GO (Gene Ontology) database, and the metabolic pathways involved in DEGs were further analyzed with reference to the KEGG database. The sequencing data are available at the NCBI (https://www.ncbi.nlm.nih.gov/bioproject/PRJNA895491/) under the accession number PRJNA895491 on 29 October 2022.

### 4.6. Metabolite Extraction and Isolation

With liquid nitrogen, the blueberry fruit sample was ground into powder, and 100 mg of it was dissolved in 500 L of 80% methanol extract. It was placed in a cold bath for 5 min after complete disintegration. The supernatant was taken and passed through a 0.22 µm microporous filter membrane after centrifugation at 4 °C (at a speed of 15,000× *g* for 20 min). Metabolite identification was accomplished using ultra-high-performance liquid chromatography (UPLC) and tandem mass spectrometry (MS/MS) analyzers. Chromatographic separation was accomplished using a Hypesil Gold column (C18). The column temperature was 40 °C, and the flow rate was 0.2 mL min^−1^. In positive ionization mode, the mobile phase A and the mobile phase B were 0.1% formic acid and methanol, respectively, while in negative ionization mode, the mobile phase A was changed to 5 mM ammonium acetate (pH 9.0). The gradient elution program was set up with reference to the protocol settings previously published. ESI was employed as the ion source for small compounds eluted from the column, and positive and negative ion scanning modes were used for sample mass spectrometry signal acquisition, respectively. The mass spectrum parameters were referred to using the previously reported method [11].

### 4.7. Metabolomic Data Processing and Analysis

Mass spectrometry data were integrated and corrected by Compound Discoverer 3.1 (CD3.1, Thermo Fisher, Waltham, MA, USA). Functional and taxonomic annotation of identified metabolites, primarily utilizing KEGG, HMDB, and LIPID MAPS databases. The processed data were first analyzed by principal component analysis (PCA) and partial least squares discrimination analysis (PLS-DA). Then, the differential abundant metabolites (DAMs) were selected based on their contribution value (VIP), fold change (FC), and significance (P) of the difference between groups. The screening criteria were VIP > 1.0, FC > 1.5 or FC < 0.667, and *p* < 0.05 [71,72]. The KEGG database was used for functional annotation of DAMs, and the annotation results were applied to perform KEGG pathway enrichment analysis.

### 4.8. Association Analysis of Transcriptomic and Metabolomic Data

The correlation analysis of DEGs and DAMs was based on the Pearson statistical method and was primarily carried out by calculating the correlation coefficient (corr) and *p* value of DEGs and DAMs. A positive correlation was defined as a corr value greater than 0, a negative correlation as a corr value less than 0, the closer the absolute value of corr was to 1, the stronger the correlation, and a significant correlation was defined as a *p* value less than 0.05.

### 4.9. qRT-PCR Analysis

For qRT-PCR amplification, a GS AntiQ qPCR SYBR Fast Mix (Genesand Biotech Co., Ltd., Beijing, China) and a QuantStudioTM 3 System real-time system were employed. The following were the reaction conditions: 95 °C for 10 s, 60 °C for 30 s, for a total of 45 cycles; 95 °C for 15 s; 60 °C for 1 min; and 95 °C for 15 s. The GAPDH gene was chosen as the reference gene [73], and the 2^−ΔΔCt^ value method was used to calculate the relative expression of the related genes. The relevant primers used in this study are shown in Table S5.

### 4.10. Statistical Analysis

All experiments included at least three replicates. All data were analyzed by ANOVA using SPSS 25.0 statistical software (IBM SPSS, Inc., Chicago, IL, USA). A Tukey test or *t*-test was employed, and differences were considered statistically significant at *p* < 0.05.

## 5. Conclusions

In summary, we performed physiological, transcriptomic, and metabolomic investigations on different blueberry fruits to uncover the mechanisms driving the variability in sugar concentration between blueberry cultivars. DEGs and DAMs associated with carbohydrate metabolism were identified in blueberries. The findings revealed that: (1) the active substance level in ‘Anna’ fruit was low, but the sugar content was high, and the antioxidant system was active, which could be employed to maintain the balance of ROS metabolism in the fruit; (2) ‘Anna’ fruit increased sugar content by activating three critical metabolic processes (starch and sucrose metabolism, glycolysis/gluconeogenesis, and the pentose phosphate pathway); (3) sugars in ‘Anna’ fruit play important roles in regulating auxin and brassinosteroid signaling; (4) eight genes from MYB, AP2/ERF, bHLH, WRKY, and MDAS-box family transcription factors were significantly enriched in blueberry fruit, which were closely related to sugar response. However, the functions of the newly identified DEGs and the regulatory roles of transcription factors need to be further studied. This study provides new insights into understanding the synthesis of carbohydrates affecting blueberry fruits and will be useful for improving fruit quality in future breeding or cultivation efforts.

## Data Availability

The data and materials supporting the conclusions are contained within the article and the Supplementary Materials. The raw sequence data are available at the NCBI (https://www.ncbi.nlm.nih.gov/bioproject/PRJNA895491/) under the accession number PRJNA895491 on 29 October 2022.

## Supplementary Materials

The following supporting information can be downloaded at: https://www.mdpi.com/article/10.3390/ijms25010293/s1.
