# Supplementary material for: Transcriptomic and Metabolomic Profiling Reveals the Variations in Carbohydrate Metabolism between Two Blueberry Cultivars
Source: Int J Mol Sci. 2023 Dec 25;25(1):293. doi: 10.3390/ijms25010293 (PMC10778917; doi:10.3390/ijms25010293)
Supplement: Supplementary file 1 [file ijms-25-00293-s001.zip › supplementary figures and supplementary table captions.pdf]

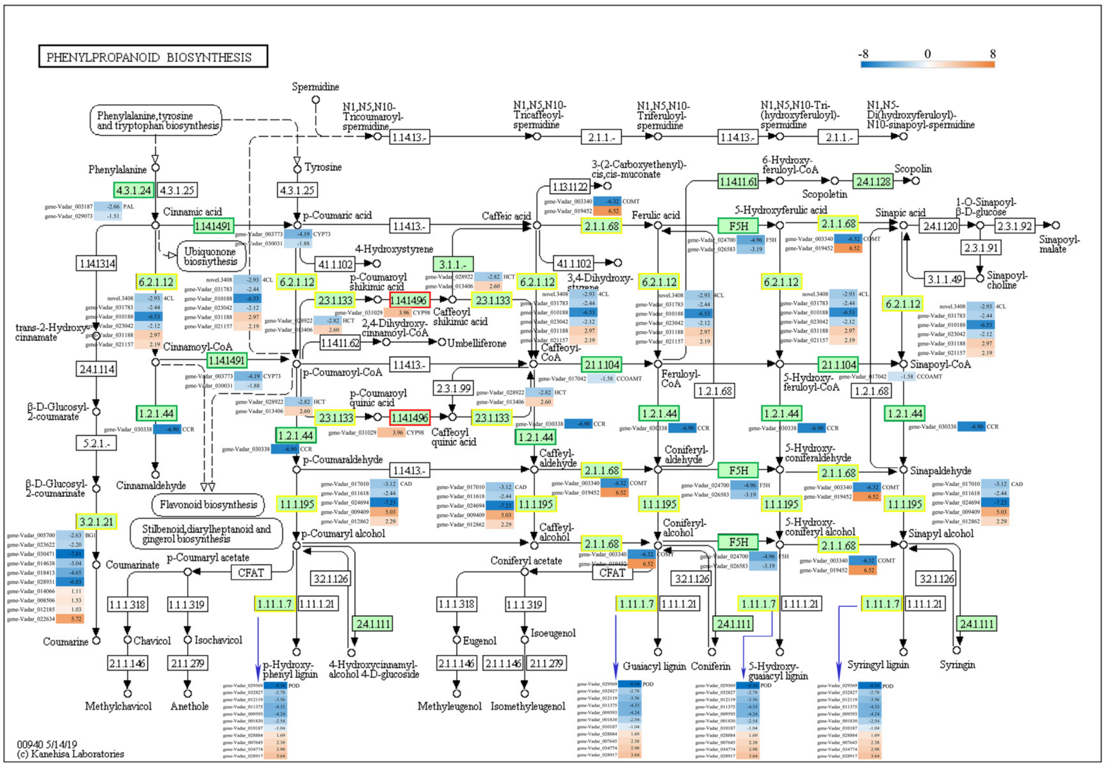

Figure S1 Heatmap of DEGs involved in the phenylpropanoid biosynthesis pathway.

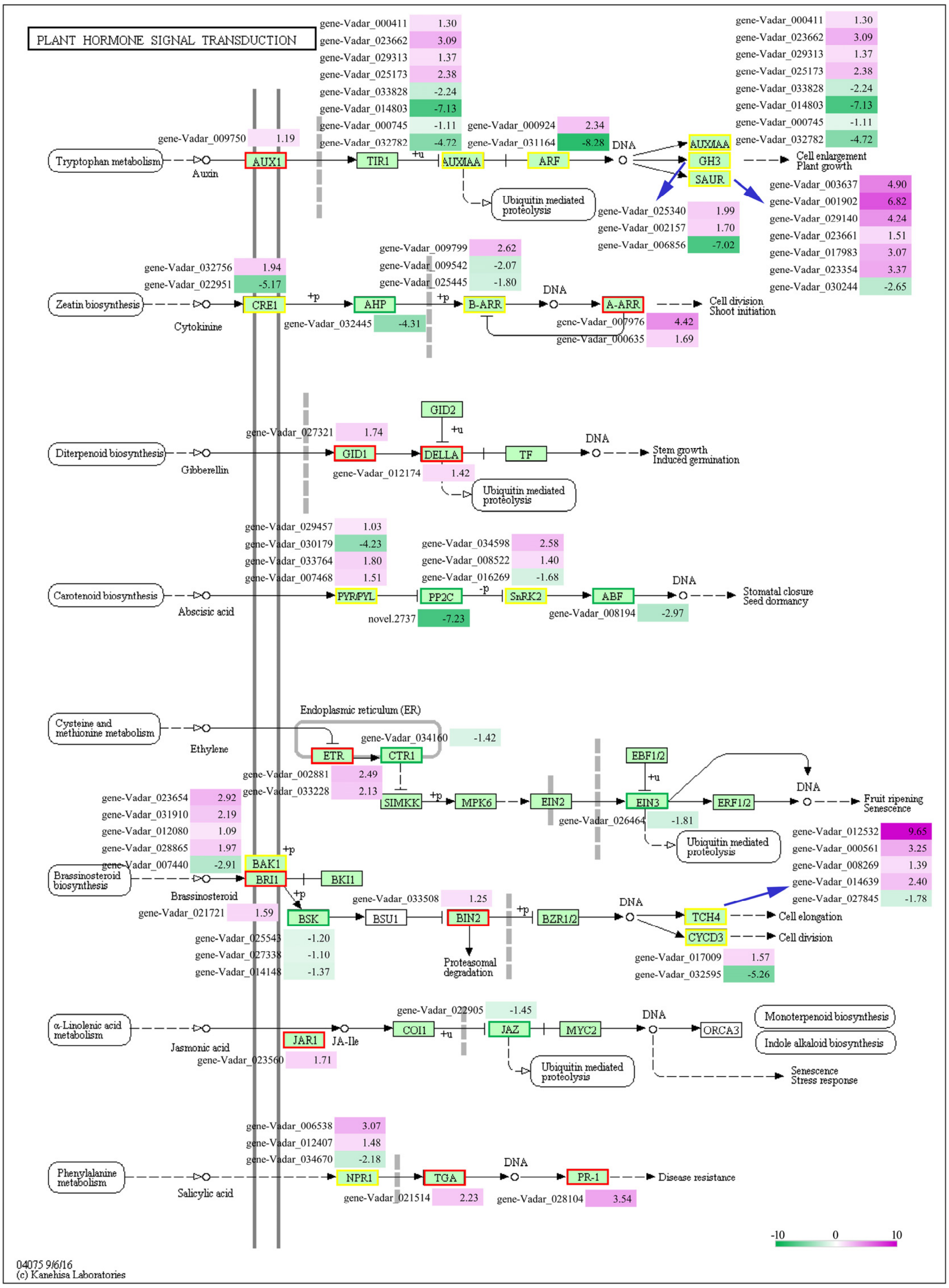

Figure S2 Heatmap of DEGs involved in the hormone signal transduction pathway.

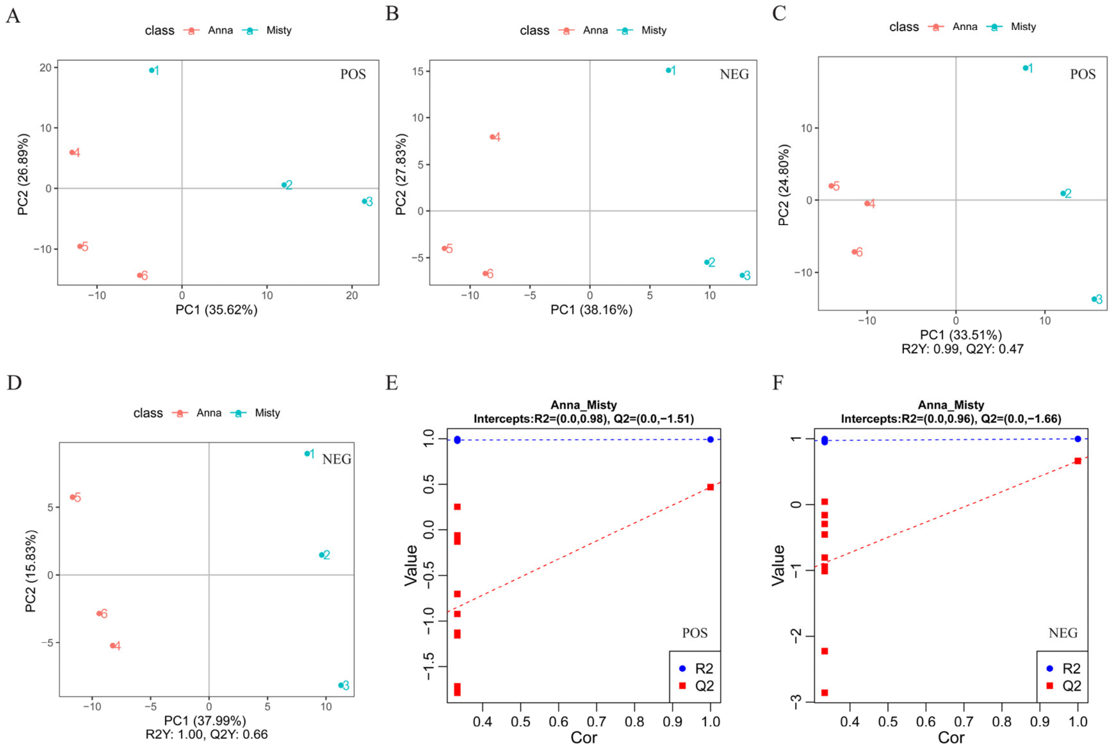

Figure S3 Multivariate statistical analysis of the metabolic profiles of the two samples ('Anna' and 'Misty'). (A-B) Principal component analysis (PCA), (C-D) Partial least squares data analysis (PLS-DA) analysis, (E-F) Permutation test results for the PLS-DA mode.

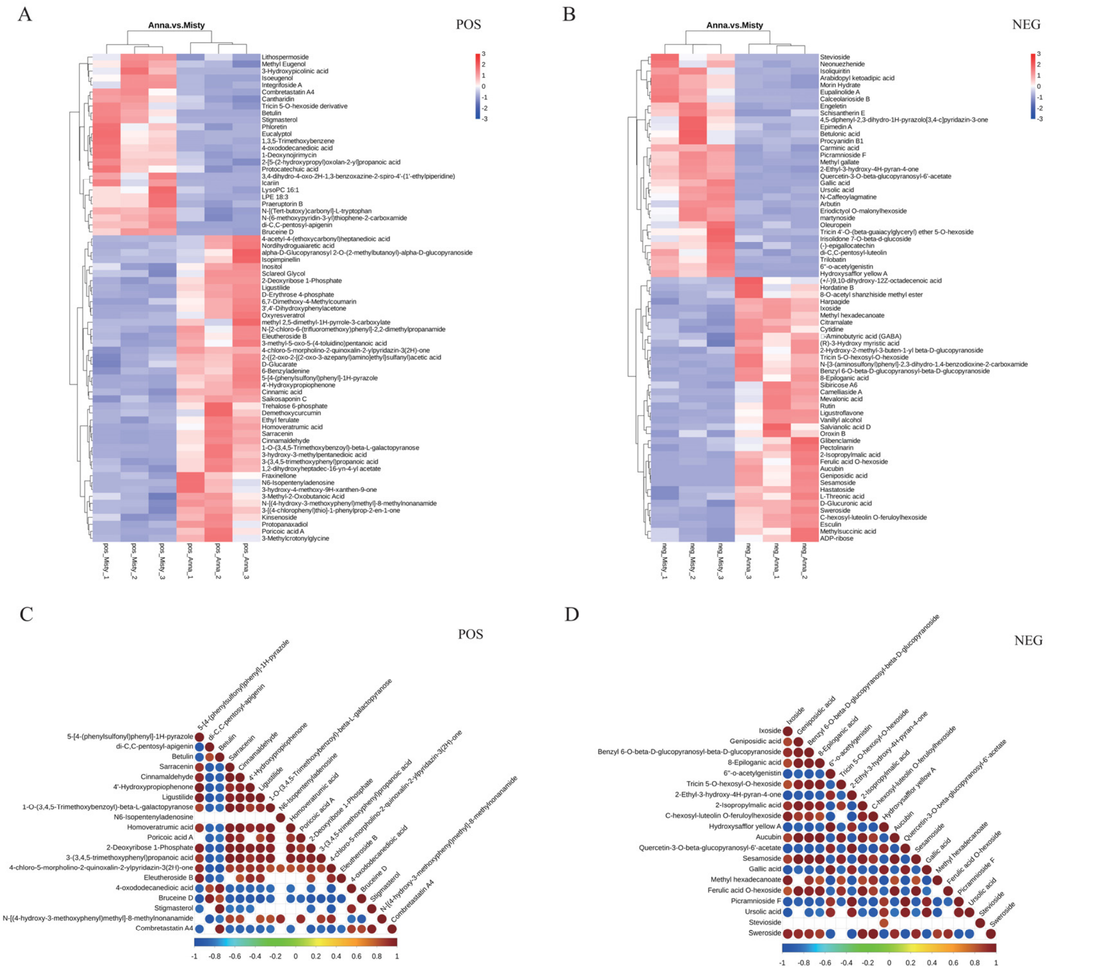

Figure S4 (A-B) Heatmap of DAMs of blueberry fruit between 'Anna' and 'Misty'. (C-D) Correlation analysis of the top 20 DAMs.

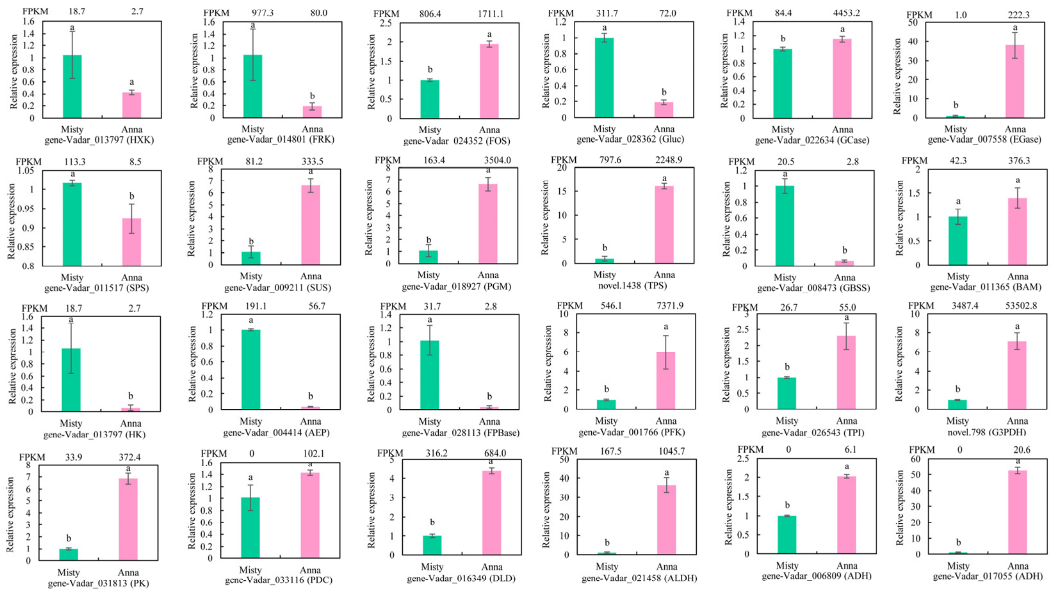

Figure S5 qRT-PCR confirmation of the expression levels of 24 DEGs from RNA-Seq. The FPKM values obtained from the RNA-Seq data are shown at the top of each image. Different lowercase letters represent statistically significant differences ($P < 0.05$) between 'Anna' and 'Misty'.

Supplementary table captions

Table S1 A summary of the genome mapping results.

Table S2 Details about the DEGs involved in the top 20 TF families.

Table S3 Detailed information on ten TFs and six DEGs involved in the carbon metabolism network that had $\log_2$ (fold change) values larger than six.

Table S4 Metabolites identified in all samples.

Table S5 Sequences of primers used for qRT-PCR.
